# The effect of hydroxycamptothecin and pingyangmycin on human squamous cell carcinoma of the tongue

**DOI:** 10.3892/ol.2013.1109

**Published:** 2013-01-07

**Authors:** PENG CHEN, BING LIU, MING HU

**Affiliations:** 1 Department of Oral and Maxillofacial Surgery, General Hospital of PLA, Beijing 100853;; 2 Department of Stomatology, General Air Force Hospital of PLA, Beijing 100036, P.R. China

**Keywords:** hydroxycamptothecin, pingyangmycin, Tca8113 cell, telomerase, apoptosis

## Abstract

The purpose of this study was to test hydroxycamptothecin (HCPT) and pingyangmycin (PYM) for their ability to inhibit the squamous cells of tongue carcinoma (Tca8113 cells). The effect of these compounds was tested using the MTT assay *in vitro*, clonogenic assays, flow cytometry, morphological observation, telomeric repeat amplification protocol (TRAP), transplantation of tumors into athymic mice and TUNEL staining. Treatment with HCPT and PYM, alone or in combination, inhibited the tumor cells and showed a greater inhibition when the drugs were combined. The cloning efficiency of Tca8113 cells was decreased. The microstructure and cell cycle of the cells changed significantly as a result of treatment. Telomerase activity was significantly inhibited in a time-dependent manner. By appearing to promote apoptosis, the drugs demonstrated a significant level of inhibition of the tumor cells in an athymic mouse model, promoting prolonged survival. HCPT and PYM have a marked cytotoxic effect on Tca8113 cells which is improved when used in combination.

## Introduction

Carcinoma of the tongue is characterized by a malignancy with rapid and invasive growth and is the most common type of oral and maxillofacial malignant tumor. Metastasis to the lymph nodes on the neck occurs frequently (1,2). In addition to surgery, chemotherapy is another important treatment method (3,4). These malignant tumors are typically treated by multi-drug combinations (5,6).

Pingyangmycin (PYM) is produced by *Streptomyces pingyangensisn* (S.P.) and has antitumor roles in the cell cycle without specificity. The mechanism of PYM is understood to introduce breaks into DNA which makes it a broad-spectrum antibiotic with anticancer potential. By preferentially acting on the dividing phase of cells with fast metabolisms, it is able to destroy tumor cells preferentially. Disturbing the metabolism of tumor cells results in apoptosis, degeneration and necrosis. PYM has a clear chemotherapeutic effect for carcinoma of the tongue (7,8), but recurrence and metastasis present challenges in certain cases. Furthermore, severe and irreversible side-effects, such as pulmonary fibrosis, have been shown to occur with large dosages (9).

Hydroxycamptothecin (HCPT), an alkaloid, is distilled from tree roots, bark or fruit. This type of tree is unique to China and belongs to the Nyssaceae family. HCPT is a cell cycle-specific botanical drug. It is able to inhibit DNA topoisomerase II (TOPO II) ligating the broken ends of DNA, causing DNA breaks and preventing the synthesis of RNA (10,11). HCPT acts directly during the S phase and prevents progression in the G_2_/M phase (12). HCPT exhibits a marked effect on tumor cells with low toxicity and a wide spectra. It has been reported to potently inhibit several tumor cell lines and transplanted tumor cells and has mainly been applied to glandular epithelium cancers in the head and neck, particularly adenoid cystic carcinoma.

It has been shown that PYM has a reasonable potency in oral squamous carcinomas with low toxicity. There have been no reports of HCPT being used for the treatment of oral squamous carcinoma or its combined usage with PYM. In the present study, HCPT and PYM were used in combination to strengthen the chemotherapeutic effect and decrease the toxicity of the treatment of squamous cell carcinoma of the tongue as modeled in cell lines in a mouse model. Although preliminary, these data suggest there is promise for future clinical studies of this combined therapy in humans.

## Materials and methods

### Materials

HCPT was provided by HSFY Pharmaceutical Industries (Hubei, China). PYM was provided by Taihe Pharmaceutical Industries (Tianjin, China). The MTT agent was purchased from Sigma Chemical Company (St. Louis, MO, USA). DMSO was purchased from Chemical Reagents Company (Beijing, China). The Telomerase PCR-ELISA kit was obtained from Boehringer (Mannheim, Germany). A TUNEL apoptosis detection kit was obtained from Boster Company (Wuhan, China). The Tca8113 cell line was obtained from the Ninth People’s Hospital, Shanghai Second Medical University (China). BALB/C nude mice (6 weeks old, 20 g average weight) were supplied by the laboratory animal research center of the General Hospital of PLA (Beijing, China). This study was approved by the Ethics Committee of the General Hospital of PLA.

### Cell culture

Tca8113 cells cultured in RPMI-1640 supplemented with 100 U/ml streptomycin, 100 U/ml penicillin and 10% FCS were incubated at 37°C in a 5% CO_2_ humidified atmosphere. The cells were digested by 0.25% parenzyme, washed with cold PBS and counted in parallel, then collected and resuspended in PBS, to prepare for inoculation.

### Nude mice

A total of 20 BALB/C nude mice were randomly assigned into four groups, each with five mice.

### Inhibition of cell growth

To test the effect of the drugs on cell growth, Tca8113 cells were expanded and harvested in the exponential growth phase. The cells were digested by 0.25% parenzyme to make a unicellular suspension. The cell density was adjusted and cells were inoculated in three 96-well plates. Each well was inoculated with 2×10^5^ cells/0.1ml and incubated at 37°C in 5% CO_2_, with saturated humidity. According to the experimental objectives, four treatment groups were prepared the next day: i) HCPT group: each well was dosed with 50 *μ*l HCPT solution of varying drug concentrations (final concentrations, 10, 32, 100 and 1,000 ng/ml), then an additional 50 *μ*l fresh culture was added; ii) PYM group: each well was dosed with 50 *μ*l PYM solution of varying drug concentrations (final concentrations tested, 32, 100, 320 and 10,000 ng/ml), then an additional 50 *μ*l fresh culture media was added; iii) combined medicine group: a total of 50 *μ*l HCPT and PYM of various concentrations were added to each well in combination; and iv) blank group: cells were cultivated without the addition of drugs under the same culture conditions described previously. After 92 h, 20*μ*l MTT (0.5%) was added for 4 h to each well in each of the four groups. After removing the supernatant, 150 *μ*l DMSO was added to resolve the crystals and determine the OD_490nm_ with a spectrophotometer according to the manufacturer’s instructions. Using the blank group to establish a 100% survival signal, the relative ODs were calculated for each group. Microsoft Excel software was used to draw a curve of the dosage effect. From this curve, the 30% cell growth inhibition concentration (IC_30_) was calculated for HCPT, PYM and the combination of the two by graphical means. This was used to select the drug concentration with the best IC_30_s, which had a limited impact on cell survival. The IC_30_s of HCPT and PYM were used for additional experiments.

### Cell cloning efficiency

The double-deck agar culture method was used in 24-well plates. Each group had four parallel wells, with 600 Tca8113 cells either treated as described previously or not treated and all were suspended in 1 ml 0.3% agar in culture media. Physiological saline (1 ml) was added into the surrounding eight wells. These cells were cultivated under normal conditions for two weeks. After two weeks, an inverted microscope was used to count the number of colonies and from this the cloning efficiency was calculated according to the following formula: cloning efficiency = number of clones / number of inoculated cells × 100%.

### Cell ultrastructure

Cell specimens were prepared for four days with either the blank treatment or IC_30_ concentrations of the drugs. These were prepared for use with a JEM-2000EX transmission electron microscope to observe the cell ultrastructure.

### Cell cycle of Tca8113

Cells were treated as described for four days, 1×10^6^ cells were collected for each group and 70% precooled alcohol was used to create single cell suspensions. These were stored at 4°C prior to FACS analysis. Before analysis, the cells were pelleted and the supernatant was discarded. Propidium iodide (PI) was used to dye the cells for 30 min, cell lumps were filtered out through a 300 screen mesh and the DNA density of the cells was read via flow cytometry.

### Telomerase activity assay

After 92 h of compound treatment, Tca8113 cells were prepared with telomerase extracting buffer. Based on the TRAP-PCR-ELISA method improved by Kim and Wu (13) to measure the telomerase activity, the assay was performed according to the kit instructions. After processing the lysates, the A_450_ was measured. The negative control group was protein extracted from Tca8113 cells after a 65°C-heat shock. The positive control group was provided by the kit. The experiment was repeated three times to produce an average value. If the change in A_450_ between an experimental group and the negative control group was >0.2, this indicated that the sample was telomerase-positive.

### Nude mouse Tca8113 cell transplantation tumors

All mice were injected subcutaneously in the right sciatic nerve with 0.2 ml 1×10^7^ Tca8113 cells/ml per nude mouse. The animals underwent chemotherapy 10 days after implantation. Four mice were randomly assigned to each treatment group and were then treated with physiological saline, PYM, HCPT or PYM/HCPT. Mice were injected with 0.2 ml physiological solution for each of the four treatment conditions, 0.05 mm from the base of the tumor. The physiological solution was administered twice each week and the drugs were administered continuously for five weeks. The dosages were as follows: PYM 4.16 mg/kg, HCPT 1.09 mg/kg, PYM/HCPT 1.00/0.35 mg/kg, respectively. The gross tumor volume was measured twice each week and was used to calculate the tumor control rate (a, long diameter; b, short diameter). Gross tumor volume = 0.5×b^2^; tumor control rate = 1 − (T_1_−T_2_) / (C_1_−C_2_) ×100, where T_1_ and T_2_ represent the average tumor volume at the beginning and end for the treatment group and C_1_ and C_2_ represent the average tumor volume at the beginning and end for the control group, respectively. The observed survival time of the athymic mice with tumors in each group was recorded.

### Apoptotic features of nude mouse Tca8113 cell transplantation tumors

When the nude mice died, a specimen was obtained for each athymic mouse tumor. The biopsies were dried, paraffin-embedded and serial sections were prepared on glass slides coated with poly-lysine. From these sections, one was randomly selected from the sections of each specimen, so that each treatment group was represented by five tumor samples which were dyed for a TUNEL assay. The sections were observed under a 20× objective lens. Five visual fields were randomly selected from each section and in each the total cell number and number of apoptotic cells was counted, in order to calculate the proportion of apoptotic cells. Those cells showing amethyst granules in the cell nucleus were positive for apoptosis.

### Statistical analysis

The differences between each group were compared using the simplex factor analysis of variance. P<0.01 was considered to indicate significant differences. Analysis was performed using the SPSS 11.0 statistical package.

## Results

### HCPT and/or PYM inhibit Tca8113 cells

To assess the potential anticancer effects of HCPT and PYM, the two drugs were first tested in a cell culture system with Tca8113 cells. *In vitro* HCPT and PYM exhibited significant growth inhibitory effects on Tca8113 cells. A greater inhibition occurred with the combination of the two compounds (Table I). After 96 h of treatment with PYM, HCPT and PYM+HCPT, the IC_30_s were determined to be 416 ng/ml, 109 ng/ml and 100+35 ng/ml, respectively.

### HCPT and PYM decrease Tca8113 cell cloning efficiency

Since the two compounds appear to be toxic to Tca8113 cells, we hypothesized that this would result in a decreased cloning efficiency in these cells. To test this, Tca8113 cells were allowed to grow on soft agar for two weeks in the presence or absence of HCPT and PYM. The cloning efficiency of the untreated group on Tca8113 cell was 31.57%, while it was 15.92, 11.46 and 4.18% for PYM, HCPT and PYM/HCPT groups, respectively. Significant differences were observed among the groups (P<0.01).

### PYM and HCPT alter the ultrastructure of Tca8113 cells

**Figure 1:**
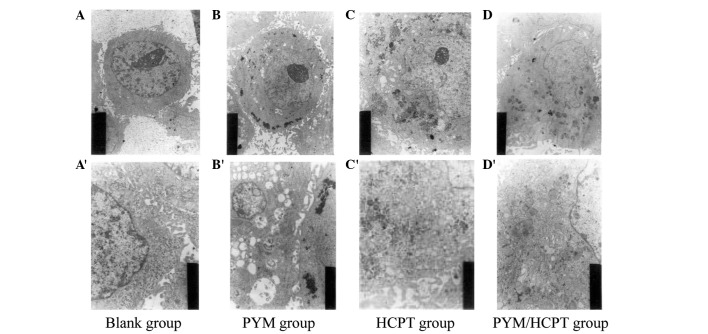
Ultrastructure of Tca8113 cells: (A,A′) blank group; (B,B′) PYM group; (C,C′) HCPT group; (D,D′) PYM/HCPT group. (A,B,C,D) Magnification, 3,000×1.45; (A′,B′,C′,D′) Magnification, 10,000×1.45. HCPT, hydroxycamptothecin; PYM, pingyangmycin.

To understand the cytotoxic effects of these compounds on the Tca8113 cells, the changes to the cells as a result of treatment were observed. The effects on the ultrastructure of Tca8113 cells are shown in Fig. 1. Compared with the control group, cells treated with PYM exhibited the following changes: an elevated nucleus to cell size ratio, multinucleation, shrinking of the nucleolus, reduction in the number of intracytoplasmic organelles and ribosomes, increased number of lysosomes and glycogen granules and different sizes of cytoplasmic vesicles. The HCPT treatment group also exhibited significant changes compared with the control, including increased cell sizes, apocytes and swollen nuclei. There were no clear changes to the nucleolus sizes or cytoplasmic vesicles, while the cytoplasmic organelles decreased sharply in number. The number of ribosomes was also observed to be reduced. The endoplasmic reticulum expanded and the number of lysosomes and glycogen granules increased significantly. Cytoplasmic vesicles of different sizes formed, even forming cellular bridges in one place. For the PYM/HCPT group, the increase in lysosome volume was more significant, with more formation of cytoplasmic vesicles. The majority of other characteristics were similar to the other treatment two groups.

### HCPT and PYM alter distribution of the cell cycle

What is known about the mechanism of the two compounds suggests that they cause DNA damage, so it was expected that treatment with these compounds would disrupt the cell cycle. To test this, unsynchronized Tca8113 cells were treated as previously with HCPT and/or PYM and then assessed by FACS analysis. The two compounds significantly altered the distribution in the cell cycle. Table II shows the effect on the cell cycle of Tca8133 cells.

### HCPT and PYM inhibit telomerase activity in Tca8113 cells

The telomerase activity of Tca8113 cells following treatment with HCPT and/or PYM was tested as described previously and the results are shown in Table III. The two drugs inhibit this activity compared with the positive control.

### Effect on the growth of Tca8113 cell transplantation tumors in nude mice

To test the effect of the compounds on a growing tumor, Tca8113 cells were transplanted into nude mice. The transplanted tumors were treated with the compounds as described previously and the tumor volume was measured. The changes in transplanted tumor volume of the blank and experimental groups is shown in Table IV. The nude mice of the blank group all died by day 48, at which point the survival rate of the PYM and HCPT groups was 60%, while it was 80% for the PYM/HCPT group.

### The effect on apoptosis in athymic mice with transplanted Tca8113 cells

After the transplanted mice died, the tumors were sampled for further analysis. We hypothesized that the compounds may inhibit tumor growth by increasing apoptosis and a TUNEL assay was used on the tumor samples to test this. Counts of the apoptotic cells in each treatment group showed that the proportion of apoptotic cells in the combined treatment group was significantly higher than treatment with either compound alone (P<0.01), suggesting synergy. A significant difference was also observed between treatment with each compound alone and the control (P<0.01). No significant differences were observed between the PYM and HCPT groups (P>0.05; Table V).

## Discussion

Given the challenges of treating tongue cancer, the present study evaluated the effects of HCPT and PYM on cancer cell growth. In the present study, HCPT showed a marked inhibitory effect on cultured Tca8113 tongue cancer cells, particularly when used in combination with PYM. This combined effect was expected given that the two compounds are known to have separate targets at different phases of the cell cycle. The combined application of PYM and HCPT enhanced the cytotoxic effects on the tumor cells, simultaneously enabling a decrease in the dosage of the two drugs which reduced the side-effects. These effects were tested and confirmed in several assays.

First, the effects of these compounds cultured Tca8113 tongue cancer cells *in vitro* were evaluated. HCPT exhibited an inhibitory effect on the cell line and this inhibition was improved when combined with PYM. In addition to this cytotoxic effect, it was observed that these compounds reduced cell cloning efficiency and increased the proportion of cells in S to G_2_ phase and multinucleation. Clear changes in the cell shape, decreased numbers of cell organelles, increased lysosomal volumes, cell degeneration and reduced telomerase activity were also observed, as well as the inhibition of the growth of transplantation tumors in athymic mice which resulted in an increased survival rate and increased apotosis.

A clonal colony formation experiment is an efficient way to detect the proliferation ability of single cell and its adaptability to the environment *in vitro*. A cell with higher cloning efficiency has greater ability to survive alone (14). Studies have shown that colony formation efficiency reflects the tumor cells’ ability to transplant in surgery (15). The present study revealed that the combination of PYM and HCPT reduced the clonal formation efficiency of Tca8113 cells. The possible clinical results of treatment with PYM and/or HCPT, are inhibition of the transformation of tumor cells at the tumor site and circulating tumor cells.

From the observed ultrastructural changes in the tumor cells, it appeared that the cellular activity was decreasing and there were signs of autophagy and programmed cell death.

In the present study, PYM, HCPT and combined treatment at IC_30_ were able to reduce the telomerase activation of cells, with the best results from the combination. This showed that inhibition decreases the telomerase activity and replication of cells. The growth inhibiting and cytotoxic effects of PYM and HCPT on tumor cells may be associated with inhibition of telomerase activity. The presumed mechanism of inhibition of telomerase activity may result from the inhibiting DNA synthesis or destroying the integrity of the DNA (16–19). The two chemicals combined increased the inhibition of the cells, promoting the inhibition of telomerase activity. Further study is necessary to better understand the mechanism of this inhibition.

Zhu *et al* reported that telomerase activity is correlated with the cell cycle phase (20). Typically, telomerase activity increases gradually after the cells enter into the G_1_/S period and reaches its highest at the S phase. During the G_2_/M phase there is no telomerase activity. However, there has been dispute about whether telomerase activity is limited to specific phases of the cell cycle. In the present study, there was no significant difference in the S to G_2_/M proportion of cells between the PYM group and the control cells. The proportion of the S to G_2_/M cells decreased significantly in the HCPT group compared with the PYM group without a significant difference being observed in the telomerase activity between these two groups. This is indicative of a connection between telomerase activity and the cell cycle. Analyzing the experiments associated with telomerase activity and cell phase, we propose that the compounds reduced not only the proportion of S to G_2_/M cells but also the telomerase activity. In this experiment, these specific and non-specific cell cycle inhibitors were administered simultaneously, which highlighted the synergy of these compounds. Further research should address the connection between telomerase activity and the cell cycle.

PYM or HCPT were used separately and in combination to treat transplanted Tca8113 tumors in athymic mice. All three treatment conditions inhibited the tumor growth and the combined treatment showed the greatest increase in survival. From this, we concluded that PYM and HCPT have inhibitory effects on tongue cancer and that this effect may be improved by using the drugs in combination. In addition, using the two compounds at a lower dose decreased the toxicity. One possible reason for this synergy is that PYM and HCPT have separate targets, as demonstrated by the impact on the cell cycle phase. PYM affected tumor cells in all phases, while HCPT sensitized cells in the S phase. The combined effect of the compounds allowed the overall treatment to overcome the shortcomings of each. Due to the low clinical efficiency of chemotherapy drugs, a large number of normal cells are usually killed while attempting to target tumor cells. This toxicity leads to serious side-effects as the dose of compounds is increased to target the tumor. The various mechanisms of chemotherapies result in harmful side-effects which harms different target organs. The combined application of PYM and HCPT decreased the dosages of each, and achieved or even exceeded the expected effects based on the separate application of each while avoiding the general toxicity.

The advantage of the TUNEL method is that it is able to detect the early period of apoptosis at a cellular level by marking the apoptotic cells (21,22). The present study used a TUNEL kit to demonstrate that the rate of apoptosis in the combined treatment group was significantly higher than that of either of the separate treatment groups (P<0.01). In addition, there was a marked difference between the two separate treatment groups and control group (P<0.01). There was no significant difference between the two separate treatment groups (P>0.05). This suggests that the two medicines accelerate cell apoptosis by separate mechanisms, resulting in a more potent effect when used in combination. Inducing the apoptosis of tumor cells is the standard mechanism of chemotherapy. The combined treatment with PYM and HCPT enhanced the apoptosis of tumor cells, resulting in improved inhibition of tumor growth. The combination of PYM and HCPT triggered apoptosis signals at different cell cycle phases and targeted the signals by different means to kill tumor cells. Specific mechanisms of action are not yet known for either compound. Further research is required to understand how these compounds act to inhibit tongue cancer.

In conclusion, combined chemotherapy with PYM and HCPT exhibited a marked effect on oral squamous carcinoma. Further research may reveal more effective dosing schemes for the clinical application of this combination chemotherapy.

## Figures and Tables

**Table I:** Effect on survival rate of HCPT and PYM in Tca8113 cells, as indicated by MTT assays after 96 h of treatment (mean, %).

HCPT (ng/ml)	PYM (ng/ml)				
	0	32	100	320	10,000
0	100.00	91.32	82.76	73.54	40.07
10	89.97	88.75	90.07	65.54	38.32
32	75.43	80.65	72.78	61.02	35.47
100	72.75	71.98	54.32	53.31	32.43
1,000	42.67	38.78	33.79	30.43	19.69

HCPT, hydroxycamptothecin; PYM, pingyangmycin.

**Table II:** Effect on growth cycle of Tca8113 cells treated with HCPT and/or PYM.

	Proportion of Tca8113 cell cycle phase (%)		
Group	G_1_ phase	S phase	G_2_ phase
Blank	69.2	21.8	9.1
PYM	79.9	14.0	6.2
HCPT	21.7	34.8	43.5
PYM/HCPT	39.0	39.4	21.5

HCPT, hydroxycamptothecin; PYM, pingyangmycin.

**Table III:** Effect on telomerase activity of Tca8113 cells following treatment with HCPT and/or PYM.

Groups	A_450_ (mean ±SD)
Positive	1.73±0.04
Negative	0.12±0.02
Blank	1.89±0.03
PYM	0.77±0.02
HCPT	0.82±0.02
PYM/HCPT	0.53±0.03

Significant differences were observed in the blank, PYM, HCPT and PYM/HCPT groups (P<0.01). HCPT, hydroxycamptothecin; PYM, pingyangmycin.

**Table IV:** Effect of treatment with PYM, HCPT or PYM/HCPT on transplanted tumor growth in nude mice.

	Gross tumor volume (mm^3^, mean ± SD)		
Groups	Pre-therapy	Post-treatment	Rate of depression tumor growth (%)
Blank	121±34	2452±456	-
PYM	108±23	1356±342	45±18.2
HCPT	119±29	1456±406	41±16.1
PYM/HCPT	123±22	634±145	74±14.7

The change in the gross tumor volume in each of the four treatment groups between pre-therapy and post-treatment is shown (n=5). No significant differences were observed in any of the groups prior to treatment (P>0.05). Significant differences were observed between pre-therapy and post-treatment (P<0.01) in all groups. Significant differences were observed between the PYM/HCPT, PYM and HCPT groups (P<0.01). HCPT, hydroxycamptothecin; PYM, pingyangmycin.

**Table V:** Percentage of apoptotic cells in all groups (n=5).

Group	Percentage (mean ±SD)
Blank	5.7±2.1
PYM	18.3±5.4
HCPT	21.6±3.2
PYM/HCPT	57.4±5.3

HCPT, hydroxycamptothecin; PYM, pingyangmycin.
